# Substrate Temperature Dependent Microstructure and Electron-Induced Secondary Electron Emission Properties of Magnetron Sputter-Deposited Amorphous Carbon Films

**DOI:** 10.3390/ma12162631

**Published:** 2019-08-19

**Authors:** Jie Li, Xingkang Yi, Wenbo Hu, Buyu Gao, Yongdong Li, Shengli Wu, Shu Lin, Jintao Zhang

**Affiliations:** Key Laboratory for Physical Electronics and Devices of the Ministry of Education, School of Electronic Science and Engineering, Xi’an Jiaotong University, Xi’an 710049, China

**Keywords:** amorphous carbon films, secondary electron emission, substrate temperature, surface roughness, sp^2^ bonds

## Abstract

For special instruments or equipments including particle accelerators, space microwave devices and spacecrafts, the suppression for electron-induced secondary electron emission (SEE) occurring on the component surfaces is of great significance due to a negative influence caused by SEE on their normal operations. In this paper, amorphous carbon (a-C) films were prepared on stainless-steel substrates by radio frequency magnetron sputtering, and the effects of substrate temperature (*T*_s_) and continuous electron bombardment on the microstructure and secondary electron emission yield (SEY) of a-C film were investigated in order to achieve a better inhibition for SEE. The experimental results show that a rise of *T*_s_ during the a-C film preparation is conducive to a SEY reduction and an increase of multipactor threshold due to the increases of surface roughness and sp^2^ bond content. In addition, although the SEY of a-C film has a slight increase with the rise of electron bombardment time, the a-C film sample with a lower SEY keeps its lower SEY all the time during continuous electron bombardment. The a-C film prepared at *T*_s_ of 500 °C has the lowest SEY peak value of 1.09 with a reduction of 30.6% in comparison with the stainless-steel substrate.

## 1. Introduction

Secondary electron emission (SEE) from solid materials has attracted a lot of attention due to its widespread applications in many technology fields such as night vision, microscopic analysis, satellite navigation, mass spectrum, and space exploration [1,2,3,4,5], and SEE usually plays a role of signal amplification in these applications. But in some cases, SEE may produce a negative effect on the normal operations of some special instruments or equipments. For instance, SEE generated by interaction between energetic charged particles and vacuum-chamber wall in particle accelerators leads to the formation of electron cloud, which considerably affects the stabilities of high-intensity particle beams [6,7,8]. For space microwave devices, multipactor closely related to SEE during the transportation of high-power radio-frequency signals weakens their performances and even results in a permanent damage on these devices [9,10,11]. In addition, for spacecrafts in space environment, surface charging mainly induced by SEE influences the electrostatic equilibrium, which probably causes the performance degradation and even failure of surface materials [12,13,14].

In order to mitigate these negative effects caused by SEE, it is necessary to lower the secondary electron emission yield (SEY) of material surfaces. Thus, various surface treatment techniques including coating component surfaces with low-SEY materials [15,16,17], forming grooved or porous microstructures on the component surfaces [18,19,20] and increasing surface roughnesses of special components have been extensively studied [21,22,23]. Among these techniques, coating low-SEY materials such as graphene, titanium nitride, chromium nitride, and amorphous carbon (a-C) on the component surfaces is the most attractive one due to its effective suppression for SEE phenomenon, wide applicability and great research value [24,25,26,27]. In recent decades, large amounts of related research about a-C film have been carried out because of its relatively low SEY and simple deposition process. For example, M. Alberti et al. produced an a-C film by pulsed laser deposition, and its maximum SEY is as low as 1.4 [28]. Vallgren et al. prepared an a-C film by direct current magnetron sputtering and found that the SEY rose by 10–20% after this film exposed to air for one month [29]. Larciprete et al. showed an a-C film deposited at room temperature by radio frequency magnetron sputtering could decrease the SEY of a clean copper surface, and found this beneficial effect was enhanced with the conversion of sp^3^ hybrids to six-fold aromatic domains [30].

However, the relationship between microstructures and SEE properties of a-C films in previous reports has not be studied in detail. Thus, in this paper, the effect of substrate temperature on the microstructure and electron-induced SEE properties of the a-C film prepared by radio frequency magnetron sputtering was investigated on the purpose of exploring the mechanism of microstructure influencing SEE properties and achieving a more effective suppression for SEE and better application of a-C films in various electronic components.

## 2. Materials and Methods 

**Figure 1:**
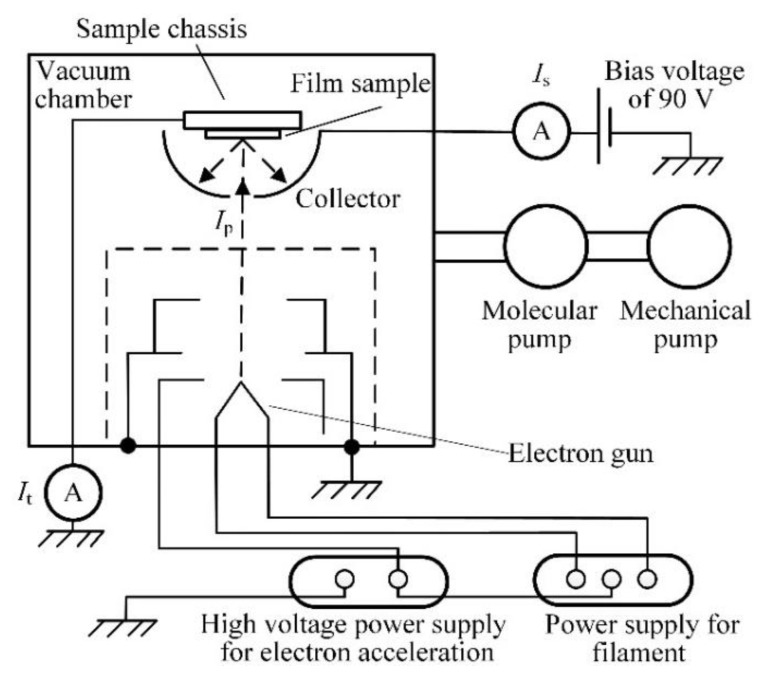
Schematic diagram of the self-designed secondary electron emission yield (SEY) measurement system.

The a-C film samples were prepared by radio frequency magnetron sputtering with a high-purity (99.99%) graphite target on stainless steel substrates under the conditions of sputtering power of 150 W, Ar gas flow rate of 30 sccm, working gas pressure of 0.11 Pa, and sputtering time of 7200 s. During the deposition process of a-C film samples, the distance and angle between the target and substrate were 150 mm and 45 °, respectively. Four a-C film samples were prepared at the substrate temperatures (*T*_s_) of 200 °C, 300 °C, 400 °C, and 500 °C to investigate the influence of *T*_s_ on their microstructures and electron-induced SEE properties. For these a-C film samples, their surface morphologies were characterized with scanning electron microscope (SEM, JSM-7000F, JEOL, Ltd., Tokyo, Japan) and atomic force microscope (AFM, Dimension Icon, Bruker, Billerica, MA, USA), and their microstructural behaviors were measured with Raman spectroscopy (LabRAM HR Evolution, Horiba Scientific, Paris, France). During the Raman measurement, the excitation wavelength of the radiation was selected to be 532 nm. In addition, their electron-induced SEE properties were estimated with a self-designed SEY measurement system shown in Figure 1. 

During the SEY measurement, every sample was mounted in the vacuum chamber (≤5 × 10^–5^ Pa), and a bias voltage of 90 V was applied to the collector with respect to the sample chassis. Primary electron current (*I*_p_) was obtained by calculation of the difference of secondary-electron current (*I*_s_) and sample chassis current (*I*_t_), and SEY was defined as the ratio of *I*_s_ to *I*_p_. For every a-C film sample, its variations of SEY with primary electron energy (*E*_p_) ranging from 50 eV to 2000 eV and bombardment time (*t*) of electron beam with *E*_p_ of 200 eV were measured. It should be pointed out the electrical current and the beam spot of electron beam utilized in the SEY measurement were 5 nA and an approximate circular area with a diameter of 1 mm, respectively.

## 3. Results and Discussion

### 3.1. Surface Morphologies of a-C Films Prepared at Different Substrate Temperatures

**Figure 2:**
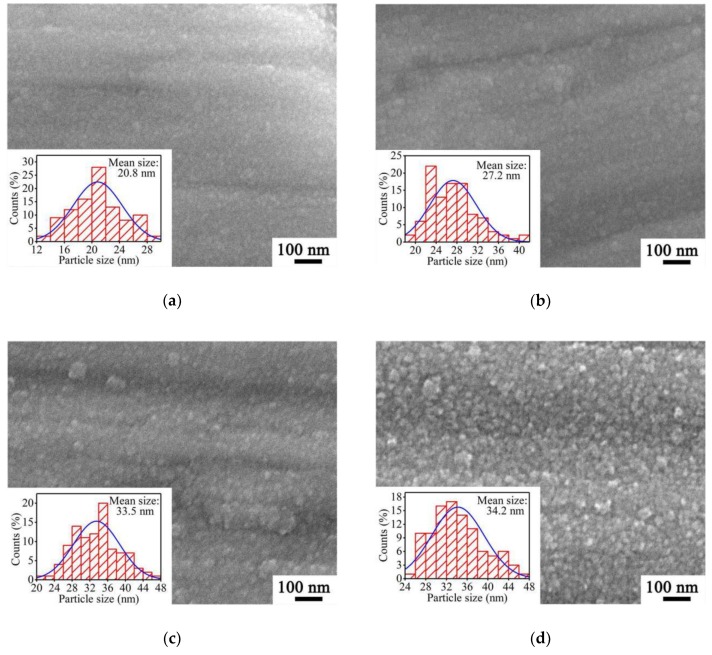
Top-viewed SEM images and their particle-diameter probability histograms of a-C film samples prepared at *T*_s_ of (**a**) 200 °C, (**b**) 300 °C, (**c**) 400 °C, and (**d**) 500 °C.

Since for a thin film material, surface morphology has an important impact on its SEY under electron bombardment, the surface morphologies of four a-C film samples prepared at different *T*_s_ were characterized by SEM. Through the SEM characterizations of cross sections, the film thicknesses of these a-C film samples were approximately in the range from 50 nm to 60 nm. Figure 2 showed the top-viewed SEM images of these a-C film samples and their particle-diameter probability histograms. 

As shown in Figure 2a, the a-C film sample prepared at 200 °C consists of large numbers of tightly arranged carbon nanoparticles with a mean size of 20.8 nm. It can be seen from Figure 2b and 2c that the mean nanoparticle sizes of a-C film samples prepared at 300 °C and 400 °C reach 27.2 nm and 33.5 nm, respectively. With *T*_s_ increasing to 500 °C, the nanoparticles of the a-C film sample shown in Figure 2d exhibit more granular and have just a slight size enlargement with the mean size reaching 34.2 nm. Based on these SEM images, it can be found the carbon nanoparticle size enlarges with the increase of *T*_s_, which reveals that a higher *T*_s_ is conducive to the growth of carbon nanoparticles in the a-C film. It is perhaps because that carbon atoms adsorbed on the substrate at a higher *T*_s_ have higher kinetic energies and are easier to migrate to form carbon nanoparticles.

**Figure 3:**
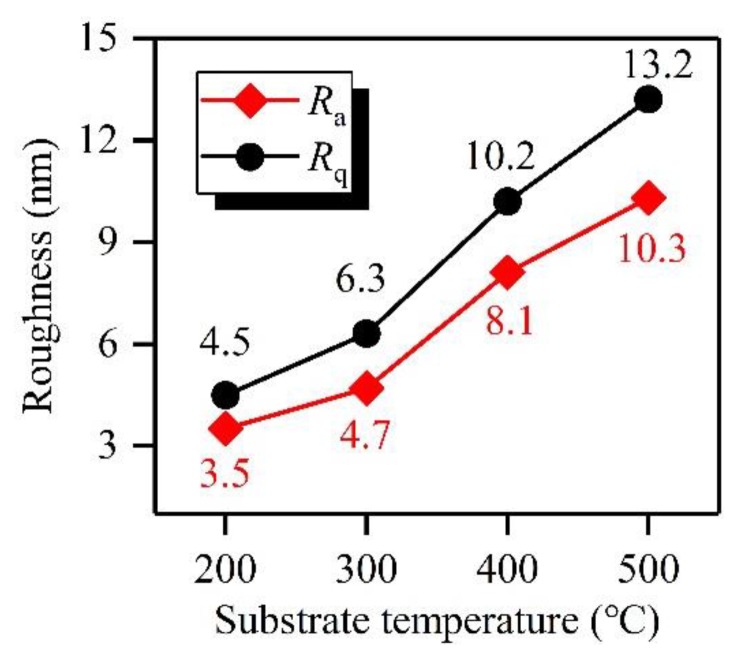
Surface roughnesses of a-C film samples prepared at *T*_s_ of 200 °C, 300 °C, 400 °C, and 500 °C characterized by AFM.

For a thin film material, surface roughness also greatly affects its SEY under electron bombardment. The thin film with a rougher surface generally has a lower SEY. Thus, the surface roughnesses of a-C film samples prepared at different *T*_s_ were measured by AFM, and the results were shown in Figure 3. Based on the experimental results, the average roughnesses (*R*_a_) of a-C film samples prepared at *T*_s_ of 200 °C, 300 °C, 400 °C, and 500 °C are 3.5 nm, 4.7 nm, 8.1 nm, and 10.3 nm, respectively, and their root mean square roughnesses (*R*_q_) are 4.5 nm, 6.3 nm, 10.2 nm, and 13.2 nm, respectively. These characterization results show that both *R*_a_ and *R*_q_ values of the a-C film exhibit a tendency of increasing with the rise of *T*_s_, which has a close relationship with the size enlargement of carbon nanoparticles shown in Figure 3.

### 3.2. Microstructural Behaviors of a-C Films Prepared at Different Substrate Temperatures

**Figure 4:**
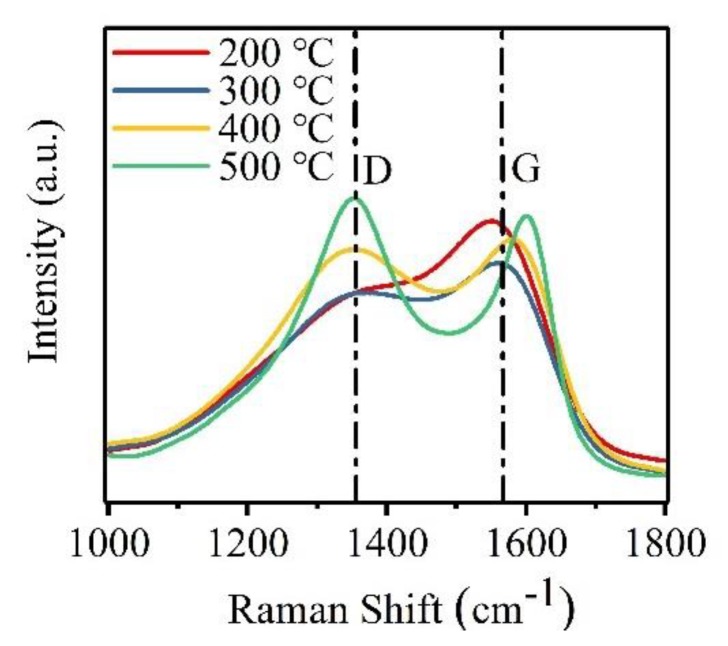
Raman spectra of a-C film samples prepared at *T*_s_ of 200 °C, 300 °C, 400 °C, and 500 °C.

**Figure 5:**
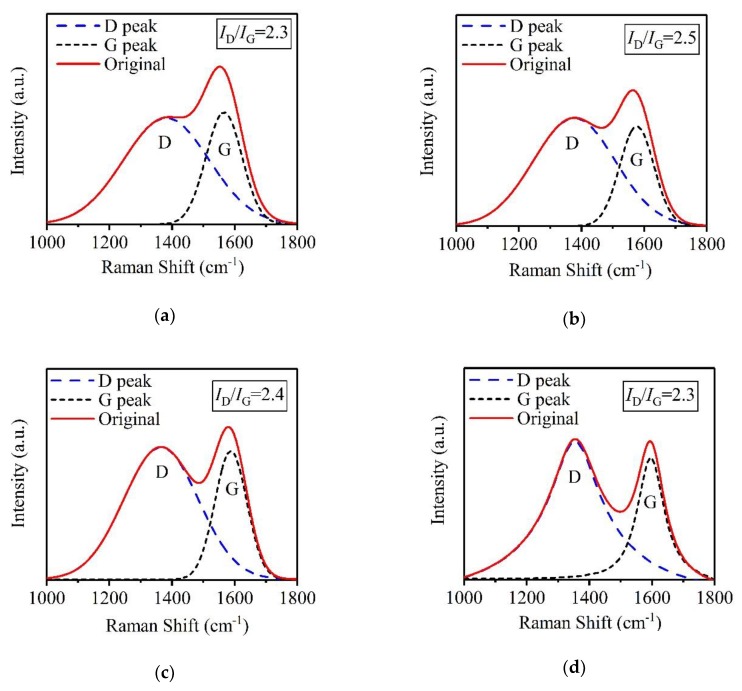
Double-peak fitting of Raman spectra of a-C film samples prepared at *T*_s_ of (**a**) 200 °C, (**b**) 300 °C, (**c**) 400 °C, and (**d**) 500 °C.

For an a-C film, microstructural behavior including sp^2^ bond content plays an important role in its SEY under electron bombardment. Generally, sp^2^ bonds in a-C films can achieve an effective suppression for SEE due to their strong scattering effects of electrons [31]. Thus, the microstructural behaviors of four a-C film samples prepared at different *T*_s_ were characterized by Raman spectroscopy, and the results were shown in Figure 4. Every original Raman spectrum takes on so-called D peak and G peak approximately located at Raman shifts of 1350 cm^−1^ and 1580 cm^−1^, respectively. The production of D peak is closely related to the breathing vibration of sp^2^ bonds, while the formation of G peak results from the stretch vibration of sp^2^ bonds [32]. It can be obviously seen from Figure 4 that with the increase of *T*_s_, the intensity of D peak strengthens and the full width at half maximum (FWHM) of G peak decreases, indicating a rise of graphite phase and a reduction of sp^3^ bond content in the a-C film, respectively [33,34]. Furthermore, as *T*_s_ increases, the G peak position of a-C film samples prepared at *T*_s_ of 200 °C, 300 °C, 400 °C, and 500 °C are approximately 1553 cm^−1^, 1567 cm^−1^, 1587 cm^−1^, and 1602 cm^−1^, respectively, which shows the G peak shifts toward a higher position, also reflecting a reduction of sp^3^ bond content [34]. As the intensity ratios of D peak and G peak are widely used to evaluate the quality of carbon films [35], it can be obtained through calculation from Figure 4 that the intensity ratios of D peak and G peak of a-C film samples prepared at *T*_s_ of 200 °C, 300 °C, 400 °C, and 500 °C are 0.8, 0.9, 1.0, and 1.1, respectively, indicating the increase of sp^2^ bond content with the rise of *T*_s_. In addition, the origin Raman spectra were decomposed into D peak curve and G peak curve based on Gaussian fitting, and the integral area ratios of D peak and G peak (*I*_D_/*I*_G_) were obtained, as shown in Figure 5. It can be seen that *I*_D_/*I*_G_ values of a-C film samples prepared at *T*_s_ of 200 °C, 300 °C, 400 °C, and 500 °C are 2.3, 2.5, 2.4, and 2.3, respectively, which exhibits the little relationship between *T*_s_ and *I*_D_/*I*_G_ value. Based on Raman characterizations, the enhancement of D-peak intensity, the increase of the intensity ratios of D peak and G peak, the shift of G peak toward a higher position and the FWHM decrease of G peak indicate that the rise of *T*_s_ promotes the graphitization of the a-C film and the simultaneous increase of sp^2^ bond content.

### 3.3. SEE Properties of a-C Films Prepared at Different Substrate Temperatures

**Figure 6:**
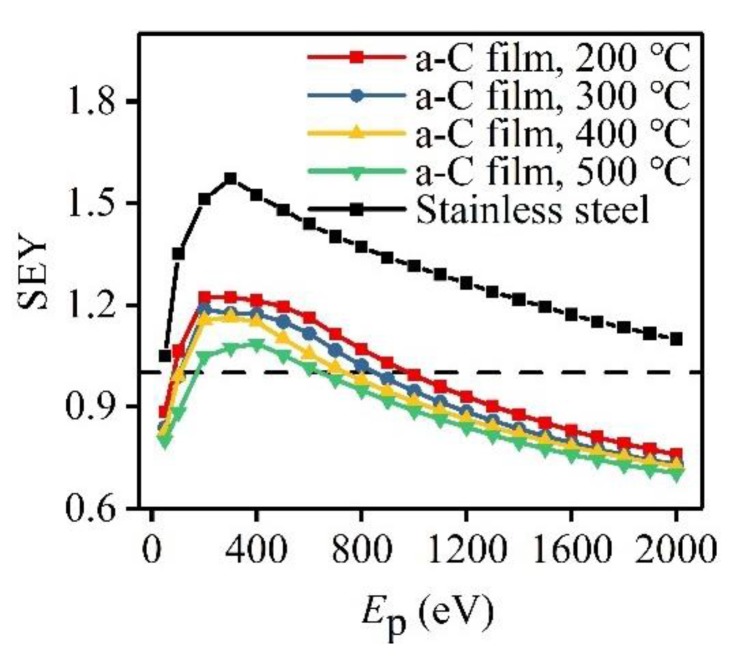
SEY-*E*_p_ curves of stainless-steel substrate and a-C film samples prepared at *T*_s_ of 200 °C, 300 °C, 400 °C, and 500 °C.

Figure 6 showed the dependences of the SEY on the primary electron energy for four a-C film samples prepared at different *T*_s_ and the stainless-steel substrate as a reference. Every SEY-*E*_p_ curve in Figure 6 exhibits the same variation tendency of firstly increasing rapidly and then decreasing gradually with the rise of *E*_p_, agreeing with the general SEE law of solid materials. It is noteworthy that every a-C film sample has a lower SEY at any certain fixed *E*_p_ than the stainless-steel substrate, which illustrates that an a-C film deposited on stainless steel can effectively reduce the SEY of this substrate. Additionally, the SEY of a-C film decreases with the increase of *T*_s_. The SEY peak values of the stainless-steel substrate and a-C film samples prepared at 200 °C, 300 °C, 400 °C, and 500 °C are 1.57, 1.22, 1.19, 1.16, and 1.09, respectively. Thus, the film sample prepared at 500 °C has the lowest SEY peak value with a reduction of 30.6% in comparison with the stainless-steel substrate. Apart from SEY, first crossover point in the SEY-*E*_p_ curve is also a major concern in the suppression for SEE. The first crossover point refers to the point where the SEY reaches 1 for the first time with the rise of *E*_p_ from 0 V in the SEY-*E*_p_ curve, and the corresponding primary electron energy of this first crossover point is called first crossover energy. It can be seen from Figure 6 that the first crossover energies in the SEY-*E*_p_ curves of a-C film samples prepared at 200 °C, 300 °C, 400 °C, and 500 °C are 81.9 eV, 104.5 eV, 107.4 eV, and 171.3 eV, respectively, which reflects that the threshold of the onset of multipactor become higher, and the suppression effect on the SEE is enhanced with the increase of *T*_s_. On basis of the microstructure characterizations, it is considered that the rougher surface and more sp^2^ bonds are two main reasons for the SEY reduction of a-C film with the increase of *T*_s_.

**Figure 7:**
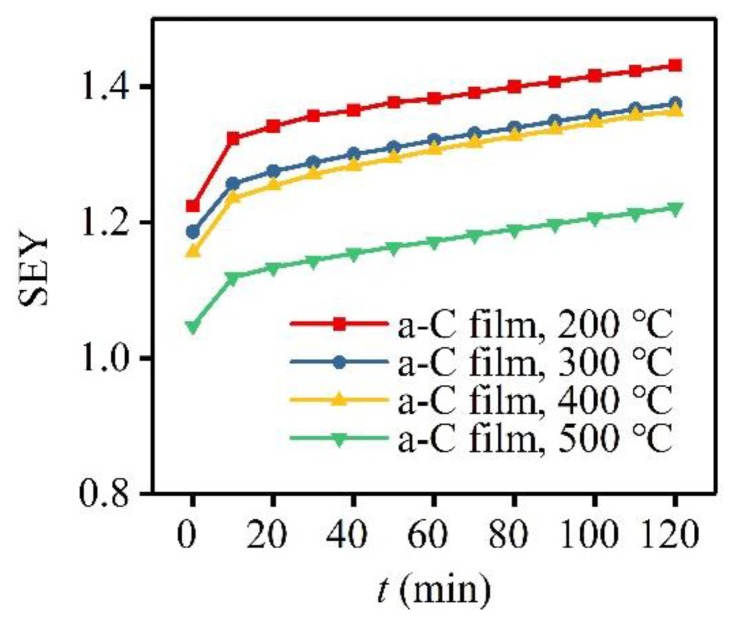
SEY-*t* curves of a-C film samples prepared at *T*_s_ of 200 °C, 300 °C, 400 °C, and 500 °C.

Based on above SEY-*E*_p_ curves, a-C films were proved to be effective in suppressing SEE, and this suppression effect becomes more obvious with the increase of *T*_s_. Then, it is of great significance to investigate the SEY variations of four a-C film samples prepared at different *T*_s_ with electron bombardment time. Therefore, SEY-*t* curves of these a-C film samples were obtained and expressed in Figure 7. Every SEY-*t* curve exhibits the same variation tendency of increasing with the rise of electron bombardment time. Through calculation, it can be obtained that all the SEYs of four a-C film samples increases by close to 17% after 120-min electron bombardment, which shows this increasing rate of SEY has little relationship with the change of substrate temperature. Among these four samples, the SEY peak value of the a-C film sample prepared at 500 °C rose from 1.05 to 1.23, just increasing by 0.18. Additionally, it should be noted that the a-C film sample with a lower SEY keeps its lower SEY all the time during continuous electron bombardment. The slight increase of SEY during continuous electron bombardment is perhaps connected with the modification of surface state of a-C film, and some relevant investigations need to be carried on further.

## 4. Conclusions

In this work, amorphous carbon films were prepared on stainless-steel substrates by radio frequency magnetron sputtering, and the influence of substrate temperature on the microstructure and secondary electron emission properties of a-C films was investigated. The experimental results show the rise of *T*_s_ during film preparation leads to the increase of surface roughness, which is related to the size enlargement of carbon nanoparticles in the a-C films. The higher *T*_s_ is also conducive to the formation of more sp^2^ bonds. In terms of the SEE properties, a-C films can effectively suppress the SEE of stainless-steel substrates, and the increase of *T*_s_ can decrease the SEY and improve the threshold of multipactor generation of the a-C film, which has a close relationship with the rougher surface and more sp^2^ bonds. It was found that the SEY of the a-C film has a slight increase with the electron bombardment time rising, but the lower SEY keeps lower all the time during the continuous electron bombardment. The a-C film prepared at *T*_s_ of 500 °C has the lowest SEY peak value of 1.09 with a reduction of 30.6% compared with the stainless-steel substrate.
